# Defective Pericyte Recruitment of Villous Stromal Vessels as the Possible Etiologic Cause of Hydropic Change in Complete Hydatidiform Mole

**DOI:** 10.1371/journal.pone.0122266

**Published:** 2015-04-07

**Authors:** Kyu Rae Kim, Chang Ohk Sung, Tae Jeong Kwon, JungBok Lee, Stanley J. Robboy

**Affiliations:** 1 Department of Pathology, University of Ulsan College of Medicine, Asan Medical Center, Seoul, Korea; 2 Department of Pathology, Myong-JI General Hospital, Goyang-si, Gyeongi-do, Korea; 3 Department of Clinical Epidemiology and Biostatistics, University of Ulsan College of Medicine, Asan Medical Center, Seoul, Korea; 4 Department of Pathology, Duke University Medical Center, Durham, North Carolina, United States of America; The University of Hong Kong, Queen Mary Hospital, HONG KONG

## Abstract

The pathogenetic mechanism underlying the hydropic change in complete hydatidiform moles (CHMs) is poorly understood. A growing body of data suggests that pericytes play a role in vascular maturation. Since maturation of villous stromal vessels in CHMs is markedly impaired at early stages, we postulated that a defect in pericytes around stromal vessels in chorionic villi might cause vascular immaturity and subsequent hydropic change. To investigate this, we examined several markers of pericytes, namely, α-smooth muscle actin (α-SMA), platelet-derived growth factor receptor-β (PDGFR-β), and desmin, in 61 normally developing placentas and 41 CHMs with gestational ages of 4–12 weeks. The ultrastructure of villous stromal vessels was also examined. Mature blood vessels from normal placentas show patent vascular lumens and formed hematopoietic components in the villous stroma. α-SMA and PDGFR-β expression in the villous stroma gradually increased and extended from the chorionic plate to peripheral villous branches. The labeled cells formed a reticular network in the villous stroma and, after week 7, encircled villous stromal vessels. In comparison, α-SMA and PDGFR-β expression in the villous stroma and stromal vessels of CHMs was significantly lower (p<0.05). Ultrastructurally, endothelial cells in villous stromal vessels in normal placentas were consistently attached by pericytes after week 7 when the vessels formed distinct lumen, whereas the villous stromal vessels in CHMs consisted of linear chains of endothelial cells, often disclosing primitive clefts without hematopoietic cells inside, and neither pericytes nor basal lamina surrounded the endothelial cells at any gestational age studied. This suggests that pericytes recruitment around villous stromal vessels is defective in CHMs and links to the persistent vascular immaturity of the villous stroma in CHMs, which in turns leads to hydropic villi.

## Introduction

Hydropically swollen chorionic villi are typically the most prominent histological abnormality in a complete hydatidiform mole (CHM). Nowadays, due to advances in ultrasonography, moles can be suspected much earlier in pregnancy, often by the 6–8^th^ week, but at this time the vesicles are much smaller [1,2] and difficult to diagnose histologically with certainty. This indicates that hydropic change is a progressive phenomenon that develops over time.

We and others have shown that even at early gestational ages CHMs already have distinctive histological features. Blood vessels are largely abortive, lacking a patent vascular lumen or hematopoietic components. Apoptotic cells in the villous stroma are common and exceed CHMs of older gestational ages [3–7]. Together, these findings are indicative of deficient vasculogenic differentiation. In both normal and molar pregnancies, the fluid present in the villi derives from the mother’s plasma in the intervillous spaces [8,9], suggesting that the absence of a patent vascular lumen in the molar stroma may prevent vascular drainage, which in turn causes vesicular fluid to accumulate in the villous stroma. Over a number of weeks, this then leads to the formation of the cisterns grossly visible by late gestation.

We believe the stromal vascular immaturity and the hydropic change of molar villi are closely linked. As endothelial markers, including CD31 and CD34, are highly expressed in the immature stromal vessels in moles [3,10,11], it seems that the differentiation of mesenchymal cells into endothelial cells is not impaired and therefore not etiologically responsible for molar development. The current study examine an alternative possible mechanism, namely abnormal pericyte development is responsible defect in CHM.

Pericytes are contractile cells that wrap around the endothelial cells of capillaries and venules throughout the body, and they share a basement membrane with endothelial cells. Pericytes have many important functions including remodeling and maturation of blood vessels [12]. They stabilize endothelial-lined vascular tubes by depositing vascular basement membrane matrix [13–15]. They regulate blood flow [16–19] and endothelial permeability and barrier properties [20,21]. They induce tissue inhibitor of metalloproteinases (TIMPs) to block metalloproteinases-dependent regression of vascular tubes [22]. They also induce myofibroblasts to produce extracellular matrix (ECM) during fibrosis and scar formation following injury [23,24]. Pericytes are also thought as therapeutic targets for tissue regeneration since they function as progenitor cells or multipotent mesenchymal stem cells [18,25].

We hypothesized that the vascular immaturity and the hydropic change of molar villi are closely related to each other and the vascular immaturity can be caused by defect of pericytes. In this study, we have examined the expression of pericyte immunohistochemical markers, namely, α-SMA, PDGFR-β, and desmin, in the villous stroma and villous stromal vessels between normal placentas and CHMs. Further, ultrastructural studies were performed to determine whether pericytes surround the stromal vessels in normal and molar villi during the early stages of pregnancy to prove that defective pericyte recruitment in CHM is the major cause of persistent immaturity of villous stromal vessels, which manifests as an increased apoptosis of endothelial and stromal cells and a hydropic molar villi. We also looked for potential correlation between basophilic/chondroid stroma, immature stromal blood vessels, numerous stromal apoptosis, and hydropic villous stroma.

## Materials and Methods

### Ethics statement

The Asan Medical Center Institutional review board approved the study (protocol 2014–0406). In accord with Declaration of Helsinki principles, the need for participants’ written informed consent was waived.

### Case Collection

Normal placentas were obtained from women who underwent elective or therapeutic abortions at early stages of pregnancy at the Department of Obstetrics and Gynecology, Asan Medical Center and Santa Hong Clinic, Seoul, Korea. All samples were histologically examined at the Department of Pathology, Asan Medical Center, Seoul, Korea. The gestational ages were calculated from the last normal menstrual period to the date of curettage. To prevent the inclusion of early stage moles among the normal placental samples, all samples were immunostained with an antibody against P57^kip2^ (clone 57P06, 1:500 dilution, Neomarker, Fremont, CA, USA); samples reacting abnormally were excluded.

Among the early CHMs (gestational age of week 4–12), those exhibiting extensive hydropic change were excluded as hydropic villi rarely contain stromal cells or blood vessels. The remaining 41 samples were at week 4 (one), week 5 (one), week 6 (five), week 7 (five), week 8 (11), week 9 (seven), week 10 (seven), week 11 (three), and week 12 (one). The gestational ages of the 62 normal placentas were week 4 (two), week 5 (13), week 6 (14), week 7 (24), week 8 (two), week 9 (four), week 10 (one), week 11 (one), and week 12 (one).

### Immunohistochemistry

Five-μm-thick formaldehyde-fixed paraffin-embedded tissue sections were transferred onto adhesive slides and dried at 62°C for 30 minutes. Following standard heat epitope retrieval for 30 minutes in EDTA, pH 8.0 in the Benchmark automatic immunostaining device (Ventana Medical System, Tucson, AZ), we incubated the samples with primary antibodies against α-SMA (1:200, DAKO, Glostrup, Denmark), PDGFR-β (1:100, Epitomics, Burlingame, CA, USA), and desmin (1:200, DAKO). The sections were subsequently incubated with biotinylated anti-mouse immunoglobulins, peroxidase-labeled streptavidin (LSAB kit, DAKO), and 3,3'-diaminobenzidine. Harris’s hematoxylin served as the counterstain. Primary antibodies were omitted during staining of negative control samples. Blood vessels in the admixed endometrial tissue served as internal positive controls.

Marker immunoreactivity was compared for blood vessels and stromal cells in both the normal and molar chorionic villi. As moles lack a discernable chorionic plate, no comparative immunoreactivity analyses of this region with the normal chorionic plate (from which primary stem villi project vertically downward) was possible.

The staining intensity of the villous stromal cells was scored from 0 to 3 independently by two pathologists (KRK and COS) where: no reactivity (0), weak (+1), moderate (+2), and strong (+3).

### Transmission Electron Microscopy

Paraffin-embedded tissue sections of five normal placentas with gestational ages of week 6 (one), week 7 (two), week 9 (one), and week 11 (one), and five CHMs with gestational ages of week 8 (one), week 10 (two), and week 11 (two), were examined using transmission electron microscopy. To localize the small number of immature blood vessels or angiogenic cell cords in the villous stroma of CHMs, regions of interest were first identified by examining villi at low magnification. The areas containing cord-like structure of immature blood vessels composed of linearly connected endothelial cells without distinct lumen were marked on hematoxylin and eosin-stained sections, and the regions were resected from the paraffin blocks using a razor blade. To remove the paraffin, the sample was immersed in xylene, rehydrated using buffers containing a decreasing concentration of alcohol, post-fixed in osmium tetroxide for 90 min, dehydrated through a graded alcohol series, and embedded in Epon-Araldite. Semi-thin sections were cut and stained with toluidine blue-O to confirm that the areas of long endothelial cords were present. The 60–80-nm-thick slices were stained with 3% lead citrate and uranyl acetate, and examined and photographed using a transmission electron microscope (Jeol 1200 EX2, Japan).

### Statistical Analysis

Differences in the expression scores of α-SMA, PDGFR-β, and desmin between normal placentas and CHMs were tested using the Mann-Whitney U test. The correlation between the gestational age and the expression score of each of the three proteins was tested using the Pearson's or Spearman's correlation test, as appropriate. Non-parametric method (Wilcoxon rank sum test) was used to calculate *p* value for each marker between the respective groups based on gestational age and *p* value of all cases were also provided in S1 Table. All tests were two-sided, and *p* values of less than 0.05 were considered to be statistically significant. Statistical analyses were performed using Stata/IC statistical software (version 12, StataCorp Ltd, TX, USA).

## Results

### Expression of Pericyte Markers in Normal Placentas

**Fig 1:**
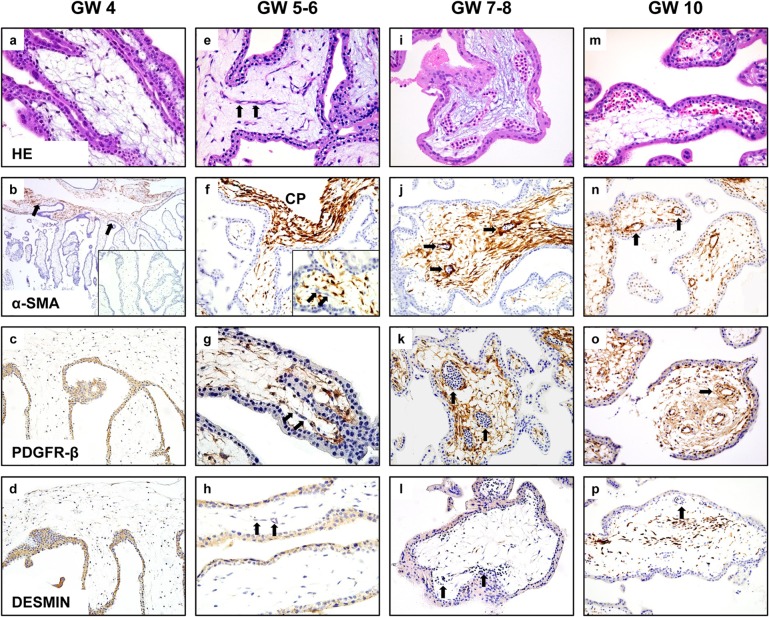
Histological changes and immunohistochemical labeling for pericyte markers in normal placentas showing significantly increased expressions of α-SMA and PDGFR-β along the vascular maturation and lumen formation. At week 4 of gestation (GW4), chorionic villi do not exhibit signs of vasculogenesis (a). Expression of α-SMA is confined to the chorionic plate (b, arrows), but not expressed in the villous stroma (b, inlet). PDGFR-β (c) and Desmin (d) are not expressed in the villous stroma. During weeks 5–6 of gestation (GW5-6), when the stroma begins to form angiogenic cell cords (e, arrows), expression of α-SMA begins to extend from chorionic plate (CP) to the villous stroma (f, inlet), but expression of α-SMA, PDGFR-β and desmin is not detected in angiogenic cell cords (arrows in f, g, h). After week 7, when the villous stroma forms mature blood vessels with lumens (i), expression of α-SMA (j and n) and PDGFR-β (k and o) markedly increases in the villous stroma forming a reticular network and encircles blood vessels (arrows in j, n, k, and o). Desmin is slightly increased in the villous stroma at week 10 (p), but is not detected in vascular structures during the entire study period (arrows in h, l, and p).

With increasing gestational age of normal placentas, the intensity of staining for α-SMA and PDGFR-β in villous stroma gradually increased, and their expression gradually extended from the chorionic plate to the peripheral villous stroma (Fig 1 and S1A Table). At older gestational ages, these markers were detected around villous stromal vessels (Fig 1 and S1B Table).

At week 4 of gestation, the stroma contained few cellular components. Signs of vasculogenesis were just beginning with angiogenic cell cords forming focally (Fig 1A). Strong α-SMA expression was largely confined to the chorionic plate (Fig 1B arrows) and did not extend to villous branches. PDGFR-β (Fig 1C) and desmin (Fig 1D) were negative or weak in the chorionic plate and peripheral villous stroma.

During gestational weeks 5–6, the time when distinct angiogenic cell cords form (Fig 1E, arrows), α-SMA expression began to extend from the chorionic plate (Fig 1F) to peripheral villous branches and the villous stroma, but was not detected in the angiogenic cell cords (Fig 1F, arrows). PDGFR-β expression remained unchanged or was slightly increased in comparison to the previous week, but was not detected in immature vascular structures of chorionic villi (Fig 1G, arrows). Desmin expression in villous stroma and vascular structure (Fig 1H, arrows) remain unchanged compared to the previous week.

During weeks 7–8 of gestation, when angiogenic cell cords began to form narrow slit-like vascular lumens and subsequently well-defined tubular lumina containing variable numbers of immature hematopoietic cells (Fig 1I), staining for α-SMA and PDGFR-β was diffuse and labeled a dense reticular meshwork in the chorionic plate and villous stroma and began to encircle vascular structure in the villous stroma (Fig 1J and 1K, arrows), making a distinct vascular structure within stroma. Desmin was negative or weakly expressed in villous stromal cells, but not in stromal blood vessels (Fig 1L, arrows).

During weeks 9 to 12, when intravascular hematopoietic components differentiated into mature and immature erythrocytes (Fig 1M), α-SMA and PDGFR-β expression remain unchanged in the villous stroma and around stromal vessels (Fig 1N and 1O, arrows) or slightly decreased in the villous stroma. The staining often labeled thick bundles in the centers of villi, which are the primordia of stem vessels. The level of desmin expression in the villous stroma was slightly increased, but it was not detected in vascular structures (Fig 1P, arrow).

### Expressions of Pericyte Markers in Complete Hydatidiform Moles

**Fig 2:**
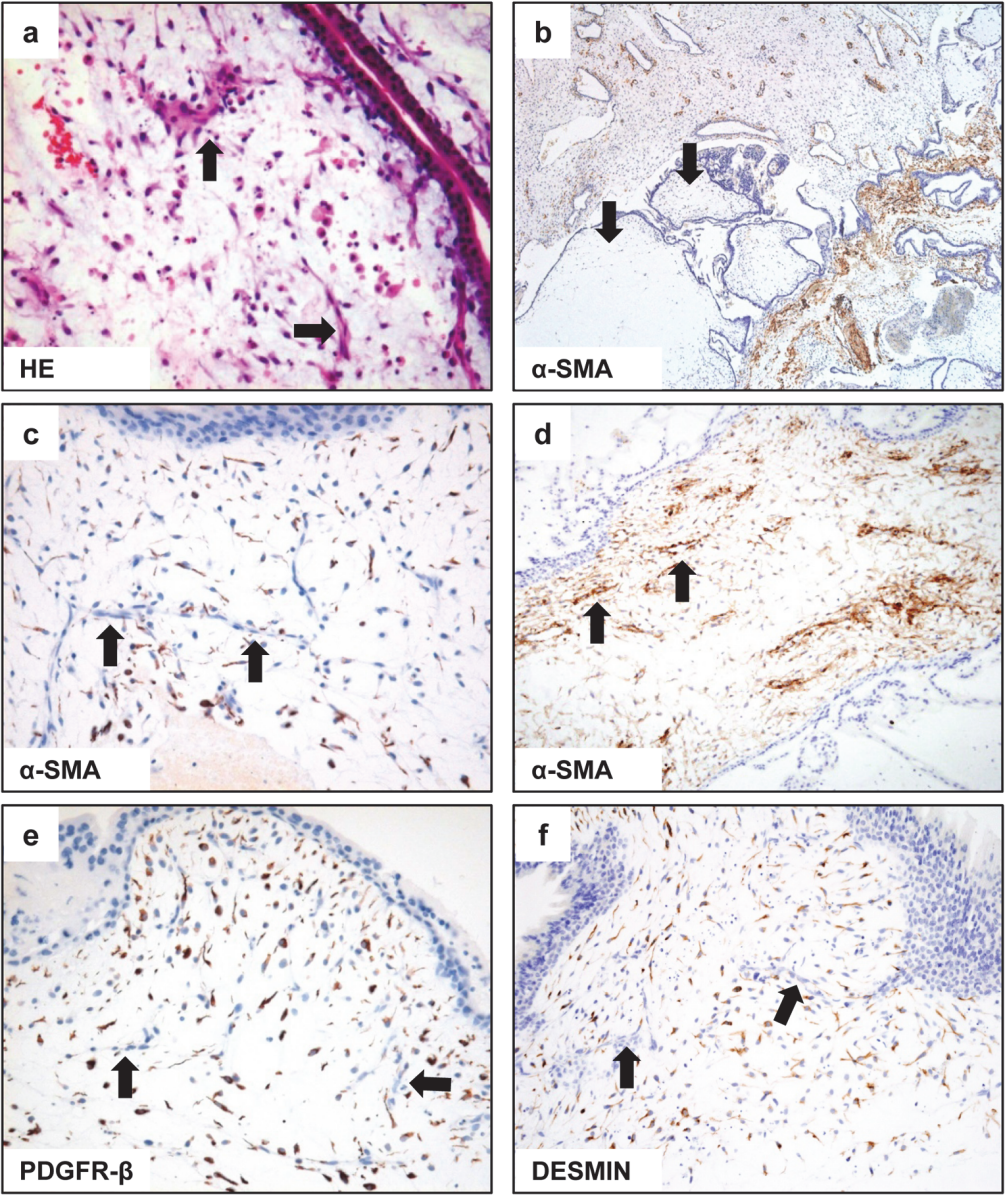
Histological changes and immunohistochemical labeling for pericyte markers in CHMs at various gestational ages. The villi have a basophilic stroma and immature vascular structures (a, arrows) that lack a distinct lumen and do not contain hematopoietic components. Note many karyorrhectic debris in vascular structure as well as in the stroma. α-SMA expression is either not expressed (b) or is weakly (c) to moderately expressed in the villous stroma, regardless of gestational age, but not in immature vascular structures (c, arrows). In one sample, α-SMA was aberrantly detected in vascular structures (d, arrows), but not detected in the surrounding villous stroma. PDGFR-β is diffusely and strongly expressed in the villous stroma in all samples, regardless of gestational age (e), but vascular expression of PDGFR-β was rarely seen (e, arrows). Desmin shows variable levels of expression in the villous stroma, but is not detected in vascular structures (f, arrows).

Most stromal blood vessels in molar villi were composed of linear cord-like structure regardless of gestational age and lacked a distinct lumen, similar to the angiogenic cell cords in normal placentas prior to week 6 of gestation (Fig 2A). Variable amounts of karyorrhectic debris were scattered in immature vascular structures (Fig 2A, arrows) and the basophilic villous stroma.

Of the 41 samples, 15 (37%) lacked α-SMA expression in the villous stroma, regardless of gestational age (Fig 2B, arrows); 22 (54%) showed weak expression (Fig 2C), and four (10%), moderate expression. α-SMA was rarely expressed in blood vessels (Fig 2C, arrows); being detected only in two samples (5%); both were 10week gestational ages and showed weak and moderate staining intensities, respectively. In one of these two samples, α-SMA was aberrantly detected only in vascular structures (Fig 2D, arrows) without forming a reticular network in the surrounding villous stroma.

PDGFR-β was diffusely and strongly expressed in the villous stroma in all samples, regardless of gestational age (Fig 2E), but only in the vascular structures of four samples (gestational ages of 6, 8, 9 and 10weeks). Desmin was expressed at variable levels in the villous stroma (Fig 2F) in all but four samples and lacked correlation with gestational age, but it was not detected in vascular structures in any samples (Fig 2F, arrows).

### Pericyte Marker Expression in Normal Placenta and CHMs

**Fig 3:**
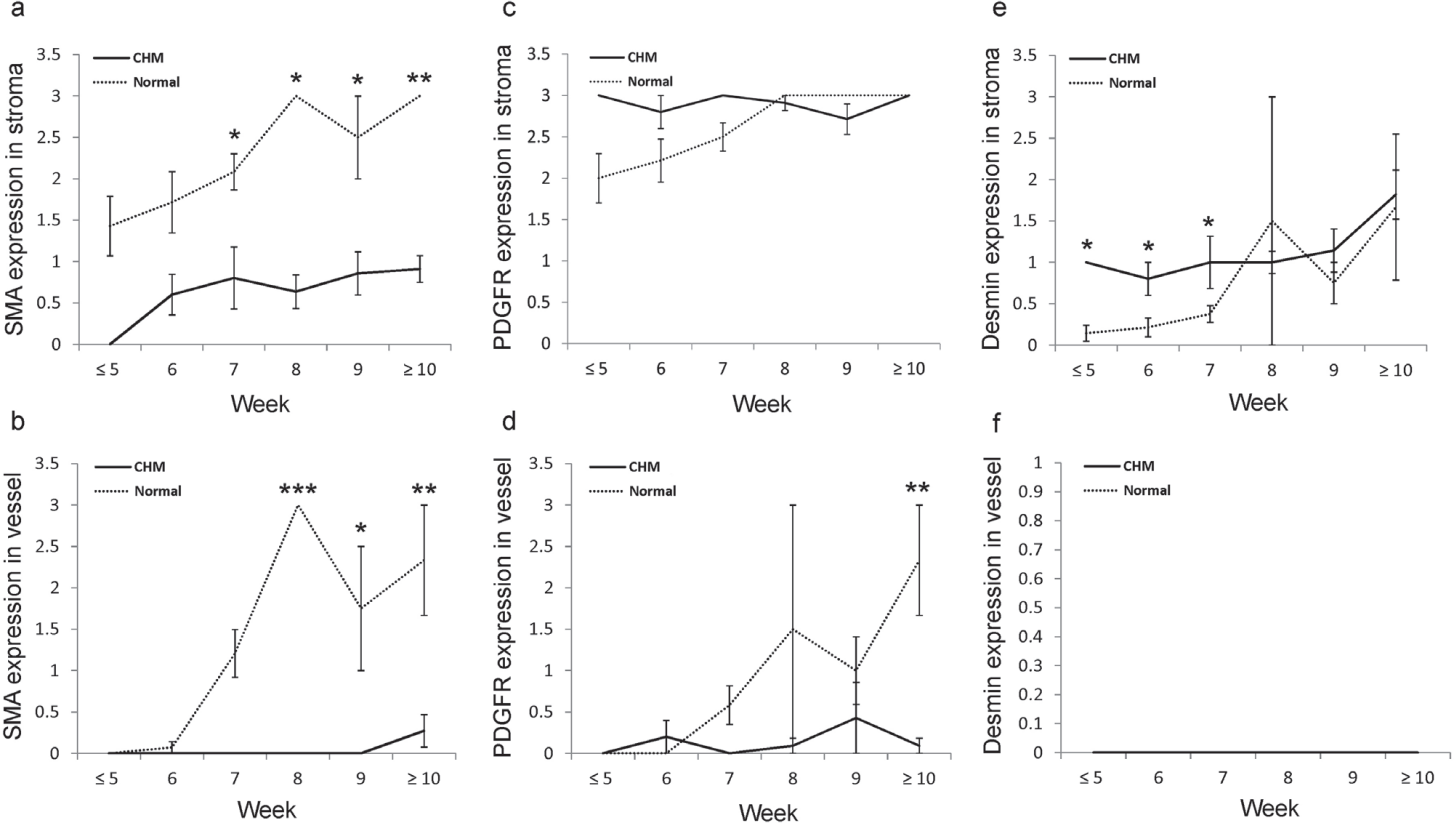
Graphical analysis of the expression levels of pericyte markers in normal placentas and CHMs. α -SMA expression in the villous stroma (a) and stromal vessels (b) is consistently lower in CHMs than in normal placentas, regardless of gestational age. (c-d), PDGFR expression in the villous stroma (c) and around stromal vessels (d) increases linearly with gestational age in normal placentas, whereas its expression does not change with gestational age in CHM, although the difference is not statistically significant. (e-f), Desmin expression in the villous stroma of normal placentas increases linearly with gestational age up to 8 weeks, whereas it does not markedly change in CHMs (e). Desmin expression is not detected in stromal vessels of normal placentas or CHMs at any gestational age (f). (*p<0.05; **p<0.01; ***p<0.001).

Composite results of all cases analyzed in the respective groups based on the gestational age are shown in Fig 3 and tabulated raw data are provided in supporting information (S1A and S1B Table). At all gestational ages, -SMA expression in the molar villous stroma (p<0.05) and molar vessels (p = 0.015) was significantly lower than in normal placentas (Fig 3A and 3B). In normal placentas, the intensity of -SMA expression in the villous stroma increased linearly with gestational age (p = 0.047). By contrast, in CHMs, -SMA expression in the villous stroma plateaued at week 6 (Fig 3A), while the -SMA expression in vascular structure was negligible when compared to that in normal placentas (Fig 3B).

PDGFR-β expression in normal villous stroma increased linearly with gestational age up to week 8 (p = 0.005), whereas its expression remain unchanged in CHMs (Fig 3C). PDGFR-β expression in normal stromal vessels increased linearly with gestational age (P = 0.011), whereas its expression was consistently lower in molar stromal vessels at all gestational ages (Fig 3D).

Desmin expression in normal villous stroma increased linearly with gestational age up to week 8, but not in CHMs (Fig 3E). Prior to week 7, desmin expression in the villous stroma was significantly higher in CHMs than in normal placentas (p = 0.039), but the difference was not observed at later stages. Desmin was not detected in stromal vessels in normal placentas and CHMs at any gestational age (Fig 3F).

### Transmission electron microscopy

**Fig 4:**
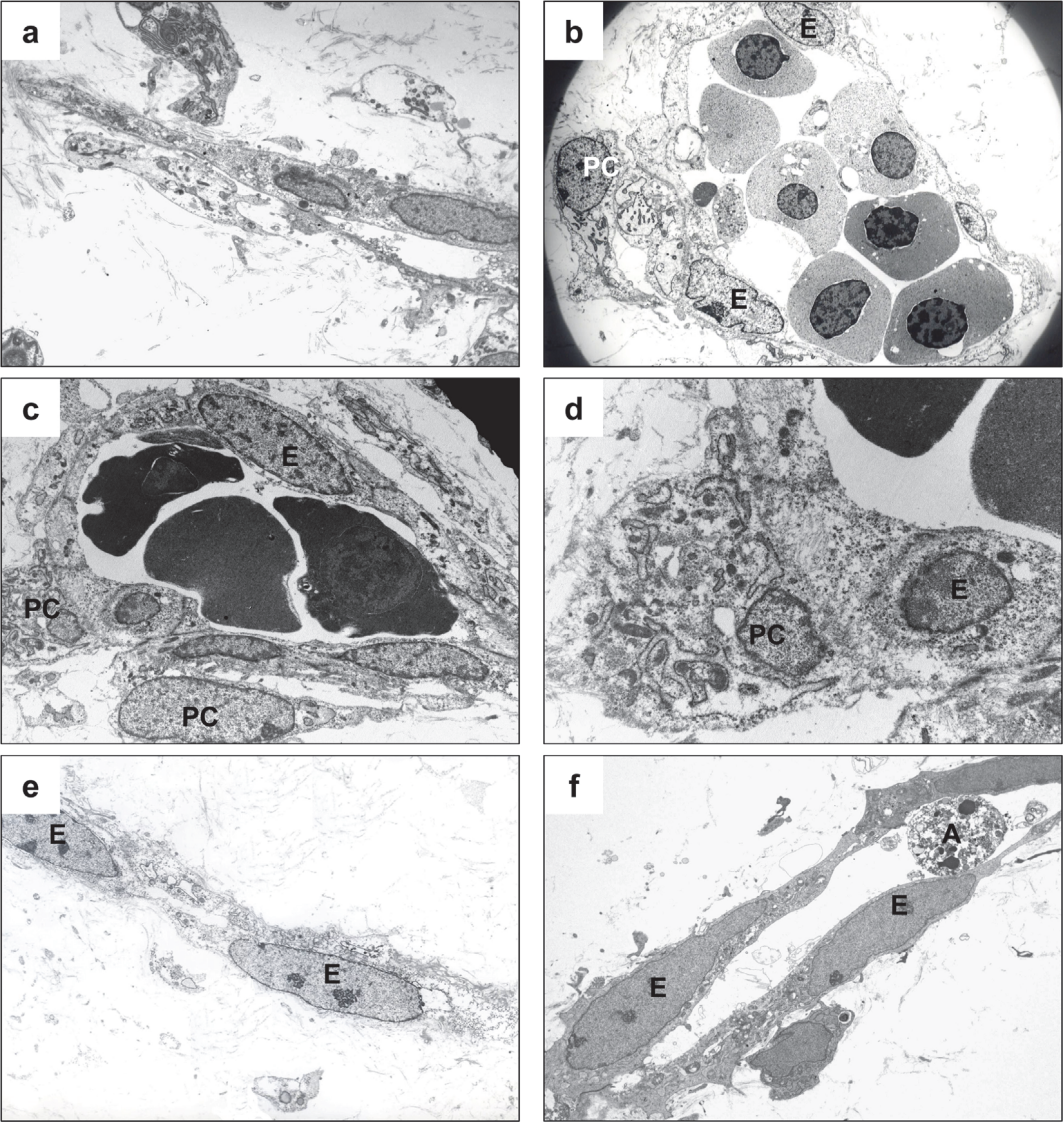
Conventional transmission electron microscopy of stromal vessels in chorionic villi of normal placentas (a-d) and CHMs (e-f) between weeks 6 and 11 of gestation. In chorionic villi of normal placentas at week 6 of gestation, immature angiogenic cell cords only contain endothelial cells, not pericytes (a). Neither a distinct vascular lumen nor a basal lamina around endothelial cells has formed. At week 7 (b), a well developed stromal vessel is lined with endothelial cells that are attached by pericytes. This vessel has a distinct lumen that contains immature hematopoietic components (b). At week 11 (c-d), mature stromal vessel contain mature or nucleated red blood cells and are lined with endothelial cells and mature pericytes. The endothelial cells and pericytes share a basal lamina. By contrast, CHMs contain immature vascular structures at week 8 (e) and week 11 (f) that are composed of degenerating endothelial cells without pericytes. Neither basal lamina nor distinct pericytes have developed. (E, endothelial cells; PC, pericyte; A, apoptotic cells).

In normal placentas at gestational week 6, angiogenic cell cords consisted of immature endothelial cells that were linearly connected (Fig 4A). These cells often had primitive clefts-like spaces between the cells, but distinct vascular lumina had not formed and scant basal lamina had been produced. Pericytes were not detected. In normal placentas at weeks 7–9 (Fig 4B), the villous stroma contained well-developed blood vessels that had distinct lumens and contained immature hematopoietic cells. The endothelial cells were attached by distinct pericytes (Fig 4B, PC), which had round to oval shaped nuclei and dilated cisternae of rough endoplasmic reticulum. The endothelial cells and adjacent pericytes shared a basal lamina. At week 11 (Fig 4C), the vascular lumen contained mature and nucleated red blood cells and showed mature endothelial cells and pericytes. The endothelial cells had tight cell junctions and contained pinocytotic vesicles. The pericytes (Fig 4C, PC) had spindle shaped nuclei and the cells were attached to the endothelial cells. The endothelial cells and pericytes shared a basal lamina (Fig 4D).

Regardless of gestational age, the vascular structures in CHMs were abnormal, always being immature and consisting of linearly arranged endothelial cell cords (Fig 4E and 4F), which resembled those in normal placentas at gestational week 6. Cleft-like spaces frequently separated the adjacent endothelial cells. At no age was there any histological evidence of vascular maturation, such as a distinct vascular lumen with microvillous processes on the luminal surface, tight intercellular junctions, pinocytotic vesicles, or a continuous basal lamina surrounding the endothelial cells. Mesenchymal cells were located close to the endothelial cells, but definite pericytes sharing basal lamina with the endothelial cells were never identified at any gestational age.

## Discussion

Pericytes, first described in 1871 [26], have until recently been ignored as important in vasculogenesis. The pericyte is now recognized to regulate vascular development, vascular tone, maturation, remodeling, stabilization and protection from regression [13–25].

The villous stroma develops from extraembryonic mesoderm at an early embryonic stage. But, pericyte origin in villous stromal vessels remains unclear. Pericytes were recently recognized as a distinct cellular component of vessel walls that share several markers in common with mesenchymal stem cells, namely CD44, CD90, CD105 and CD73 [27]. Perivascular stromal cells also express the commonly used pericyte markers, NG2 (transmembrane chondrotin sulfate proteoglycan) and PDGFR-β [27,28]. Therefore, some populations of pericytes are defined as multipotent stem cells, and they possess mesenchymal plasticity [29]. Indeed, an immunohistochemical and ultrastructural study reported that perivascular stromal cells can differentiate into both smooth muscle cells and stromal myofibroblasts in chorionic villi [30].

In the current study, the villous stromal cell differentiation into myofibroblasts was initiated at the chorionic plate in normal placenta, and gradually extended to the peripheral villous branches over time, and myofibroblastic differentiation of villous stromal cells in the normal placenta occurred prior to the differentiation of vascular smooth muscle cells and pericytes in normal placentas. The deficient expression of both stromal and vascular α-SMA in CHMs indicates defective myofibroblastic differentiation and pericyte development in the villous stroma and stromal vessels.

It is difficult to study pericytes because of their close spatial relation to vascular endothelial cells. The biochemical and physiological features of pericytes cannot be distinguished from those of vascular smooth muscle cells; both contain smooth muscle actin fibers and abundant endoplasmic reticulum. The pericytes in many different organs are morphologically, biochemically, and physiologically heterogeneous. Moreover, no single immunohistochemical marker is known to identify all pericytes in various organs because the expression of markers is dynamic and depends on the species, tissue, and developmental status. Consequently, ultrastructural observation is a standard method to confirm the pericytes by their location, but pericytes of immature blood vessels may not be developed in early embryonic period depending on the gestational weeks. Thus, combination of pericyte markers, α-smooth muscle actin (α-SMA), platelet-derived growth factor receptor-β (PDGFR-β), desmin, NG2 proteoglycan, myosin heavy chain of smooth muscle cell, caldesmon, calponin, aminopeptidase N, aminopeptidase A, and nestin have been used to identify pericyte [31,32]. In this study, ultrastructural analyses confirmed that stromal vessels in CHMs were immature and lacked pericytes, regardless of gestational age, indicating a close relation between vascular immaturity and an absence of pericytes.

We attempted to explain how the histological features of early CHM, including basophilic/chondroid stroma, immature stromal blood vessels, numerous stromal apoptosis, and hydropic villous stroma are interrelated. Our previous studies showed that CD31-positive angiogenic cell cords in molar villi lack a vascular lumen and intravascular hematopoietic components [3,4]. Blood vessels must have a lumen for blood to circulate and for component exchange. During normal vascular morphogenesis, pericytes, through their recruitment around endothelial cells, affect vascular tube maturation and stabilization events through the deposition of basement membrane matrix [14,15,33,34]. Basement membrane matrices were observed only when the endothelial cells were attached by pericytes in vivo and in vitro system [14]. In the current study, villous stromal vessels in CHMs did not have pericytes around them. Although they often had primitive cleft-like spaces between endothelial cells (Fig 4E and 4F), there was no histological evidence of vascular maturation, including microvillous processes on the luminal surface, tight intercellular junctions, pinocytotic vesicles, and basal lamina. Intravascular hematopoietic components were not identified, regardless of gestational age. These findings suggest that in CHMs, hemangioblastic stem cells do not mature into hematopoietic stem cells, and angioblasts also have a defective maturation in vasculogenesis. Why pericytes are not recruited to villous stromal vessels in CHM is still unknown. Mounting evidence indicates that cyclin-dependent kinase inhibitors of the Cip/Kip family, including p57^(Kip2)^ and p27^(Kip1)^, not only control cell cycle exit but also participate in many important cellular processes including cell fate and terminal differentiation, cell motility and migration, and cell death/survival, both in peripheral and central nervous systems [35–37]. Thus, absence of p57^Kip2^expression in CHM might have related to the lack of terminal differentiation of villous stromal component.

Recent studies have shown also that the local density of extracellular matrix determining the stiffness of the extracellular matrix regulates the rate of neovessel growth and branching in sprouting angiogenesis [12,38,39]. Basophilic/chondroid matrix in early molar stroma might be an unfavorable environment for vasculogenesis.

Endothelial cells and pericytes are thought to be the major source of TIMP-2 and TIMP-3, respectively, which protect normal vascular tubes from regression in a matrix metalloproteinase-1 (MMP-1)-, MMP-10-, and a disintegrin and metalloproteinase-15 (ADAM-15)-dependent manner [22,40,41]. For this reason, defective pericyte recruitment in CHMs may lead to caspase-dependent cleavage of molecules [42], which likely increased the number of apoptotic stromal cells.

In conclusion, this study suggest that the characteristic histological findings of CHM at early gestational stages, including basophilic/chondroid villous stroma, immature stromal vessels, a high number of apoptotic cells in the villous stroma, and hydropic change in villi, are closely related. Defective pericytes recruitment is likely a major cause of the persistent immaturity of molar villous stromal vessels, which results in an increased number of apoptotic endothelial and stromal cells and a hydropic change in molar villi.

## Supporting Information

S1 Table S1A. Tabulated raw data for α-SMA, PDGFR-β, and Desmin expression in stroma between CHM and normal placenta. S1B. Tabulated raw data for α- SMA, PDGFR-β, and Desmin expression in vessel between CHM and normal placenta.
(DOCX)
