# Supplementary material for: Defective Pericyte Recruitment of Villous Stromal Vessels as the Possible Etiologic Cause of Hydropic Change in Complete Hydatidiform Mole
Source: PLoS One. 2015 Apr 7;10(4):e0122266. doi: 10.1371/journal.pone.0122266 (PMC4388658; doi:10.1371/journal.pone.0122266)
Supplement: S1 Table — S1B. Tabulated raw data for α- SMA, PDGFR-β, and Desmin expression in vessel between CHM and normal placenta. (DOCX) [file pone.0122266.s001.docx]

**S1A Table. Tabulated raw data for SMA, PDGFR, and Desmin expression in stroma between CHM and normal placenta.**

|  | **≤5 weeks** | | |  | **6 weeks** | | |  | **7 weeks** | | |  | **8 weeks** | | |  | **9 weeks** | | |  | **≥10 weeks** | | |
| --- | --- | --- | --- | --- | --- | --- | --- | --- | --- | --- | --- | --- | --- | --- | --- | --- | --- | --- | --- | --- | --- | --- | --- |
|  | n | mean (SD) | |  | n | mean (SD) | |  | n | mean (SD) | |  | n | mean (SD) | |  | n | mean (SD) | |  | n | mean (SD) | |
| **SMA** |  |  | |  |  |  | |  |  |  | |  |  |  | |  |  |  | |  |  |  | |
| CHM | 2 | 0 | (0.00) |  | 5 | 0.6 | (0.55) |  | 5 | 0.8 | (0.84) |  | 11 | 0.64 | (0.67) |  | 7 | 0.86 | (0.69) |  | 11 | 0.91 | (0.54) |
| Normal | 14 | 1.43 | (1.34) |  | 14 | 1.71 | (1.38) |  | 24 | 2.08 | (1.06) |  | 2 | 3 | (0.00) |  | 4 | 2.5 | (1.00) |  | 3 | 3 | (0.00) |
| *P* value |  | 0.1282 | |  |  | 0.1546 | |  |  | 0.0192 | |  |  | 0.0212 | |  |  | 0.0272 | |  |  | 0.0042 | |
| **PDGFR** |  |  | |  |  |  | |  |  |  | |  |  |  | |  |  |  | |  |  |  | |
| CHM | 2 | 3 | (0.00) |  | 5 | 2.8 | (0.45) |  | 5 | 3 | (0.00) |  | 11 | 2.91 | (0.30) |  | 7 | 2.71 | (0.49) |  | 11 | 3 | (0.00) |
| Normal | 14 | 2 | (1.11) |  | 14 | 2.21 | (0.97) |  | 24 | 2.5 | (0.83) |  | 2 | 3 | (0.00) |  | 4 | 3 | (0.00) |  | 3 | 3 | (0.00) |
| *P* value |  | 0.2123 | |  |  | 0.2554 | |  |  | 0.1764 | |  |  | 0.6698 | |  |  | 0.2598 | |  |  | NA | |
| **Desmin** |  |  | |  |  |  | |  |  |  | |  |  |  | |  |  |  | |  |  |  | |
| CHM | 2 | 1 | (0.00) |  | 5 | 0.8 | (0.45) |  | 5 | 1 | (0.71) |  | 11 | 1 | (0.45) |  | 7 | 1.14 | (0.69) |  | 11 | 1.82 | (0.98) |
| Normal | 14 | 0.14 | (0.36) |  | 14 | 0.21 | (0.43) |  | 24 | 0.38 | (0.49) |  | 2 | 1.5 | (2.12) |  | 4 | 0.75 | (0.50) |  | 3 | 1.67 | (1.53) |
| *P* value |  | 0.0112 | |  |  | 0.0233 | |  |  | 0.0472 | |  |  | 0.9039 | |  |  | 0.3217 | |  |  | 0.8679 | |

CHM, complete hydatidiform mole; SD, standard deviation

**S1B Table. Tabulated raw data for SMA, PDGFR, and Desmin expression in vessel between CHM and normal placenta.**

|  | **≤5 weeks** | | |  | **6 weeks** | | |  | **7 weeks** | | |  | **8 weeks** | | |  | **9 weeks** | | |  | **≥10 weeks** | | |
| --- | --- | --- | --- | --- | --- | --- | --- | --- | --- | --- | --- | --- | --- | --- | --- | --- | --- | --- | --- | --- | --- | --- | --- |
|  | n | mean (SD) | |  | n | mean (SD) | |  | n | mean (SD) | |  | n | mean (SD) | |  | n | mean (SD) | |  | n | mean (SD) | |
| **SMA** |  |  | |  |  |  | |  |  |  | |  |  |  | |  |  |  | |  |  |  | |
| CHM | 2 | 0 | (0.00) |  | 5 | 0 | (0.00) |  | 5 | 0 | (0.00) |  | 11 | 0 | (0.00) |  | 7 | 0 | (0.00) |  | 11 | 0.27 | (0.65) |
| Normal | 14 | 0 | (0.00) |  | 14 | 0.07 | (0.27) |  | 24 | 1.21 | (1.41) |  | 2 | 3 | (0.00) |  | 4 | 1.75 | (1.50) |  | 3 | 2.33 | (1.15) |
| *P* value |  | NA | |  |  | 0.5501 | |  |  | 0.065 | |  |  | 0.0005 | |  |  | 0.0113 | |  |  | 0.0063 | |
| **PDGFR** |  |  | |  |  |  | |  |  |  | |  |  |  | |  |  |  | |  |  |  | |
| CHM | 2 | 0 | (0.00) |  | 5 | 0.2 | (0.45) |  | 5 | 0 | (0.00) |  | 11 | 0.09 | (0.30) |  | 7 | 0.43 | (1.13) |  | 11 | 0.09 | (0.30) |
| Normal | 14 | 0 | (0.00) |  | 14 | 0 | (0.00) |  | 24 | 0.58 | (1.14) |  | 2 | 1.5 | (2.12) |  | 4 | 1 | (0.82) |  | 3 | 2.33 | (1.15) |
| *P* value |  | NA | |  |  | 0.0943 | |  |  | 0.2201 | |  |  | 0.1166 | |  |  | 0.1243 | |  |  | 0.0017 | |
| **Desmin** |  |  | |  |  |  | |  |  |  | |  |  |  | |  |  |  | |  |  |  | |
| CHM | 2 | 0 | (0.00) |  | 5 | 0 | (0.00) |  | 5 | 0 | (0.00) |  | 11 | 0 | (0.00) |  | 7 | 0 | (0.00) |  | 11 | 0 | (0.00) |
| Normal | 14 | 0 | (0.00) |  | 14 | 0 | (0.00) |  | 24 | 0 | (0.00) |  | 2 | 0 | (0.00) |  | 4 | 0 | (0.00) |  | 3 | 0 | (0.00) |
| *P* value |  | NA | |  |  | NA | |  |  | NA | |  |  | NA | |  |  | NA | |  |  | NA | |

CHM, complete hydatidiform mole; SD, standard deviation
